# Supplementary material for: Multiscale computational modeling of the cardiopulmonary consequences of postnatal hyperoxia with implications for preterm-born children
Source: Biomech Model Mechanobiol. 2026 Mar 30;25(2):32. doi: 10.1007/s10237-026-02047-9 (PMC13035550; doi:10.1007/s10237-026-02047-9)
Supplement: Supplementary file 1 — Supplementary file1 (DOCX 2484 kb) [file 10237_2026_2047_MOESM1_ESM.docx]

**SUPPLEMENT for “Multiscale Computational Modeling of the Cardiopulmonary Consequences of Postnatal Hyperoxia with Implications for Preterm Born Children” in Journal of Biomechanics and Modeling in Mechanobiology**

Salla M Kim^1,2^, Filip Jezek^3^, Pim JA Oomen^1,2,4^, Gregory P Barton^5^, Daniel A Beard^3^, Kara N Goss^5,6^, Mitchel J Colebank^1,7,8^*, and Naomi C Chesler^1,2^*

1. Cardiovascular Innovation and Research Center, University of California Irvine, Irvine, California, USA

2. University of California Irvine, Department of Biomedical Engineering, Irvine, California, USA

3. University of Michigan, Department of Molecular & Integrative Physiology, Ann Arbor, Michigan, USA

4. Center for Complex Biological Systems, University of California Irvine, Irvine, California, USA

5. University of Texas Southwestern Medical Center, Department of Internal Medicine, Dallas, Texas, USA

6. University of Texas Southwestern Medical Center, Department of Pediatrics, Dallas, Texas, USA

7. University of South Carolina, Department of Mathematics, Columbia, South Carolina, USA

8. University of South Carolina, Department of Biomedical Engineering, Columbia, South Carolina, USA

* Corresponding Author Emails: [nchesler@uci.edu](mailto:nchesler@uci.edu); [mjcolebank@sc.edu](mailto:mjcolebank@sc.edu)

**Contents**

- S.1 Preclinical Data
- S.2 Circulation Model Volume Distributions and Pressure Scaling
- S.3 Nominal Parameters for Nx
- S.4 Model Variables
- S.5 Calibrated Parameters
- S.6 Additional Model Outputs
- S.7 Additional Sensitivity and Identifiability Analysis

**S.1 Preclinical Data**

The averaged data from Kumari et al. (2019) separated by condition and sex is show in **Table S1**. No significant difference was found between the male (M) and female (F) subjects for the Hx group except for RV ESV. The number of subjects in the Nx group was insufficient for statistical relevance. The average ventricular wall weight to bodyweight ratio for Nx and Hx, calculated from a separate cohort of P21 Nx and Hx rats from Kumari et al. (2019) that did not undergo hemodynamic measurements, are shown in **Table S2**. Wall weights were determined for the LV and septum combined. Here, we assume the LV comprises 2/3 of the total combined volume and surface area of the LV and septum. From **Table S1-2**, we calculated additional data used in the model as described in **Table S3**. Finally, metabolite concentrations for the Nx group (assumed identical for M and F) are from adult rats used in (Marzban et al., 2020) and are listed in **Table S4**.

**Table S1. Group Averages of Measured Data Separated by Condition and Sex**

| Metric | Units | Nx M (n = 5) | Nx F (n = 2) | Hx M (n = 7) | Hx F (n = 5) | p (Hx) |
| --- | --- | --- | --- | --- | --- | --- |
| BW | g | **50.4** | **49** | **53.9** | **52.6** | **NS** |
| T | s | **0.181** | **0.166** | **0.178** | **0.178** | **NS** |
| SV | µL | **23** | **41** | **26** | **32** | **NS** |
| LVESV | µL | **18** | **10** | **19** | **17** | **NS** |
| RVESV | µL | **23** | **26** | **19** | **29** | **0.04** |
| LVEDP | mmHg | **4.6** | **4** | **5.3** | **4.8** | **NS** |
| LVESP | mmHg | **66** | **63** | **69** | **76** | **NS** |
| RVEDP | mmHg | **3.4** | **1.75** | **3.85** | **3.8** | **NS** |
| RVESP | mmHg | **27** | **25** | **39** | **36** | **NS** |
| BW – body weight; T – time; SV – stroke volume; ESV – end systolic volume; EDV – end diastolic volume; EDP – end diastolic pressure; ESP – end diastolic pressure; LV – left ventricle; RV – right ventricle. p – p-value from a t-test between Hx M and Hx F. NS – not significantly different. | | | | | | |

**Table S2. Average Wall Weight to Body Weight Ratios (WR_avg,j_)**

|  | Nx (n=14) | Hx (N=12) |
| --- | --- | --- |
| LV + SEP | 3.8 | 3.2 |
| LV | 2/3 · 3.8 | 2/3 · 3.2 |
| SEP | 1/3 · 3.8 | 1/3 · 3.8 |
| RV | 0.86 | 1.0 |

**Table S3. Calculated Data**

| Symbol | Definition | Calculation |
| --- | --- | --- |
| HR | Heart Rate | 60/T |
| TBV | Total Blood Volume | BW · 60 · 1e-3 |
| CO | Cardiac Output | HR · SV |
| SBP | Systemic Systolic Blood Pressure | LVESP/1.05 |
| DBP | Systemic Diastolic Blood Pressure | SBP - 40 |
| W_i_ | Ventricular Wall Weight | WR_avg,i_ · BW |
| T – heart period  LVESP – left ventricular end systolic pressure  WR_avg,I_ – average ratio for wall weight and body weight  BW – body weight | | |

**Table S4. Nx Cardiac Metabolite Data**

| Symbol | Definition | Value | Unit |
| --- | --- | --- | --- |
| [ATP] | ATP Concentration | 8.0 | mM·(l cytosol water)^−1^ |
| [ADP] | ADP Concentration | 0.05 | mM·(l cytosol water)^−1^ |
| [Pi] | Pi Concentration | 1.3 | mM·(l cytosol water)^−1^ |

**A.2 Circulation Model Volume Distributions and Chamber Pressure Scaling**

Compartmental volumes of the circulation model are scaled by the total blood volume (TBV) (**Table S3**) using blood volume fractions (listed in **Table S5**) based on Beneken (1979) and Kim et al. (2023). The volume in the four vascular compartments (systemic arteries, SA; systemic veins, SV; pulmonary arteries, PA; pulmonary veins, PV) is the sum of the unstressed and stressed volumes. The unstressed volume is defined as the maximal amount of blood the compartment can hold while having zero pressure on its wall, and the stressed volume is defined as the volume of blood beyond the unstressed volume that raises the compartment pressure above zero. The unstressed volume is a fraction of the TBV, $d_{u}$, listed in **Table S5**.

We used nominal healthy pressures (denoted by a hat, $\hat{}$) for each model compartment which are used in the calculation of resistances, compartmental compliances, and chamber elastances. Nominal pressures, shown in **Table S5**, are approximated based on subject-specific EDV and ESP data (**Table S1**), and the venous pressures are adapted from Boron (2016) and Kim et al. (2023).

**Table S5. Blood Volume Fractions and Nominal Pressure Estimates**

| **Compartment** | **Symbol** | **TBV**  **Fraction (**$d_{i}$**)** | **Unstressed Volume Fraction (**$d_{i,u}$**)** | **Maximal Pressure (**$\hat{P}_{i,M}$) | **Mean Pressure (**$\hat{P}_{i,bar}$) | **Minimal Pressure (**$\hat{P}_{i,m}$) | |
| --- | --- | --- | --- | --- | --- | --- | --- |
|  |  |  |  | mmHg | mmHg | mmHg | |
| Left ventricle | LV | 0.025 | - | LVESP | - | LVEDP | |
| Systemic arteries | SA | 0.1 | 0.70 | SBP | DBP+1/3·(SBP-DBP) | DBP | |
| Systemic veins | SV | 0.45 | 0.90 | 8 | 3 | 2 | |
| Right ventricle | RV | 0.025 | - | RVESP | - | RVEDP | |
| Pulmonary arteries | PA | 0.05 | 0.40 | RVESP/1.05 | - | $\hat{P}_{PA,M}$-15 | |
| Pulmonary veins | PV | 0.35 | 0.90 | 8 | 3 | 2 | |
| LVESP, LVEP, SBP, DBP, RVESP, and RVEDP are from **Table A1** and **Table A3**. | | | | | | |  |

**S.3 Nominal Parameters for Nx**

The nominal parameter values for the multiscale model are shown in **Tables S6-10**. The asterisk (*) denotes parameters modified according to **Table 3** to represent Hx.

**Table S6. Crossbridge Kinetics Parameters**

| Symbol | Definition | Value | Unit |
| --- | --- | --- | --- |
| $k_{a}$ | Myosin-actin rate of attachment | 448 | s^-1^ |
| $k_{d}$ | Myosin-actin rate of unattachment | 305 | s^-1^ |
| $K_{\mathrm{Pi}}$ | [Pi] dissociation constant | 4.00 | mM |
| $k_{1}$ | Transition rate constant | 112 | s^-1^ |
| $k_{-1}$ | Rate of strongly-bound to weakly-bound transition | 21.3 | s^-1^ |
| $\alpha_{1}$ | Strain-dependency parameter | 10.0 | µm^-1^ |
| $k_{2}$ | Rate of ratcheting | 812 | s^-1^ |
| $k_{-2}$ | Rate of reverse ratcheting | 43.3 | s^-1^ |
| $\alpha_{2}$ | Strain-dependency parameter | 9.1 | µm^-1^ |
| $K_{D}$ | [MgADP] dissociation constant | 0.194 | µm |
| $K_{T}$ | [MgATP] dissociation constant | 0.490 | µm |
| $k_{3}$ | Myosin-actin detachment rate | 145 | s^-1^ |
| $\alpha_{3}$ | Strain-dependency parameter | 5.93 | µm^-2^ |
| $s_{3}$ | Strain-dependency parameter | 9.9e-3 | µm |
| $k_{\mathrm{on}}$ | Rate constant of Ca binding to troponin C | 101 | µM^-1^ s^-1^ |
| $k_{off, LV}$ | LV rate constant of Ca unbinding to troponin C | 724 | s^-1^ |
| $k_{off, RV}$* | RV rate constant of Ca unbinding to troponin C | 724 | s^-1^ |
| $K_{\mathrm{coop}}$ | Strength of thin filament cooperativity | 9.68 | - |
| $k_{\mathrm{SR}}$ | Rate constant of force-dependent super relax transition | 15 | s^-1^ |
| $k_{-\mathrm{SR}}$ | Reverse rate constant of force-dependent super relax transition | 50.0 | s^-1^ |
| $k_{\mathrm{force}}$ | Force-dependent rate constant of super relax transition | 1.17 | N^-1^ m^-2^ |
| $k_{\mathrm{TS}}$ | Calcium transient fraction of time for max systole | 0.1 | - |
| $k_{\mathrm{TR}}$ | Calcium transient fraction of time for relaxation | 0.3 | - |

**Table S7. Sarcomere Geometry Parameters**

| Symbol | Definition | Value | Unit |
| --- | --- | --- | --- |
| $L_{s,ref}$ | Reference Sarcomere length | 2.0 | µm |
| $L_{sc0}$ | Contractile Element Length | 1.51 | µm |
| $L_{\mathrm{thick}}$ | Length of thick filament | 1.67 | µm |
| $L_{h,bare}$ | Length of bare region of thick filament | 0.10 | µm |
| $L_{\mathrm{thin}}$ | Length of thin filament | 1.20 | µm |

**Table S8. Myofiber Force Parameters**

| Symbol | Definition | Value | Unit |
| --- | --- | --- | --- |
| $k_{passive,LV}$ | LV passive stiffness constant | 50 | mmHg µm^-1^ |
| $k_{passive,RV}$* | RV passive stiffness constant | 50 | mmHg µm^-1^ |
| $\gamma$ | Steepness constant | 7 | - |
| $k_{\mathrm{stiff},1, \mathrm{LV}}$ | LV stiffness constant due to myosin-actin interaction | 1.82e4 | mmHg µm^-1^ |
| $k_{\mathrm{stiff},1, \mathrm{RV}}$* | RV stiffness constant due to myosin-actin interaction | 1.82e4 | mmHg µm^-1^ |
| $k_{\mathrm{stiff},2,\mathrm{LV}}$ | LV stiffness constant due to working stroke of crossbridges | 4.78e4 | mmHg µm^-1^ |
| $k_{\mathrm{stiff},2,\mathrm{RV}}$* | RV stiffness constant due to working stroke of crossbridges | 4.78e4 | mmHg µm^-1^ |
| $\Delta R$ | Crossbridge displacement associated with ratcheting deformation | 0.010 | µm |
| $ƞ$ | Viscosity | 1 | mmHg s µm^-1^ |
| $K_{\mathrm{se}}$ | Series elastic element elastance | 5e4 | mmHg µm^-1^ |

**Table S9. TriSeg Parameters**

| Symbol | Definition | Value | Unit |
| --- | --- | --- | --- |
| $V_{w,\mathrm{LV}}$ | Left ventricle wall volume | eq. A1 | cm^3^ |
| $V_{w,\mathrm{SEP}}$ | Septal wall volume | eq. A1 | cm^3^ |
| $V_{w,RV}$ | Right ventricle wall volume | eq. A1 | cm^3^ |
| $A_{m,ref,LV}$ | Left ventricle midwall reference area | eq. A6 | cm^2^ |
| $A_{m,ref,SEP}$ | Septal midwall reference area | eq. A6 | cm^2^ |
| $A_{m,ref,RV}$ | Right ventricle midwall reference area | eq. A5 | cm^2^ |

**Table S10. Circulation Parameters**

| Symbol | Definition | Value | Unit |
| --- | --- | --- | --- |
| $C_{\mathrm{SA}}$ | Systemic arterial compliance | eq. A7 | cm^3^ kPa^-1^ |
| $C_{\mathrm{SV}}$ | Systemic venous compliance | 3 | cm^3^ kPa^-1^ |
| $C_{\mathrm{PA}}$ | Pulmonary arterial compliance | eq. A7 | cm^3^ kPa^-1^ |
| $C_{\mathrm{PV}}$ | Pulmonary venous compliance | eq. A7 | cm^3^ kPa^-1^ |
| $R_{\mathrm{SA}}$ | Systemic arterial resistance | eq. A8 | kPa s cm^-3^ |
| $R_{\mathrm{PA}}$ | Pulmonary arterial resistance | eq. A8 | kPa s cm^-3^ |
| $R_{m}$ | Mitral valve resistance | 0.1 | kPa s cm^-3^ |
| $R_{a}$ | Atrial valve resistance | 0.0333 | kPa s cm^-3^ |
| $R_{t}$ | Tricuspid valve resistance | 0.1 | kPa s cm^-3^ |
| $R_{p}$ | Pulmonary valve resistance | 0.0333 | kPa s cm^-3^ |

The ventricular wall volumes, $V_{w,i}$ (cm^3^) where $i=LV, SEP, RV$, were calculated from the approximate ventricular wall weights, $W_{i}$ (**Table S2**), assuming a cardiac wall density of 1.055 g/mL (Gheorghe et al., 2019; Vinnakota & Bassingthwaighte, 2004), such that

| $V_{w,i}=\frac{1}{1.055}\cdot W_{i}.$ | (S1) |
| --- | --- |

We assumed that of the combined LV + SEP wall volume, the LV is 2/3 and the septum is 1/3, and we assumed the LV and septum have the same wall thickness. Given EDV and an assumption of spherical geometry, we calculated the wall thickness $h_{i}$. First, we calculated the inner chamber radius, such that

| $r_{\mathrm{LV}}=\left( \frac{3\cdot\mathrm{LVEDV}}{4\pi} \right)^{\frac{1}{3}} \text{and}\text{ } r_{\mathrm{RV}}=\left( \frac{3\cdot\mathrm{RVEDV}}{4\pi} \right)^{\frac{1}{3}}.$ | (S2) |
| --- | --- |

Then, we calculated the wall thickness for the LV and septum,

| $h_{\mathrm{LV}},h_{\mathrm{SEP}}=\left( \frac{3}{4\pi}{\cdot(V}_{w,LV}+V_{w,SEP}+LVEDV) \right)^{\frac{1}{3}}-r_{\mathrm{LV}},$ | (S3) |
| --- | --- |

and we assumed the RV wall thickness, $h_{\mathrm{RV}}$, is half $h_{LV+SEP}$.

Finally, given the inner chamber radius and wall thickness, we calculated the midwall radius and ultimately the midwall reference surface area. The midwall radius is

| $r_{m,LV}=r_{\mathrm{LV}}+\frac{h_{m,LV}}{2} \text{and}\text{ } r_{m,RV}=r_{\mathrm{RV}}+\frac{h_{m,RV}}{2}.$ | (S4) |
| --- | --- |

Finally, the midwall reference surface areas were calculated as

| $A_{m,ref,LV+SEP}=4\pi r_{m,LV}^{2} \text{and}\text{ } A_{m,ref,RV}=4\pi r_{m,RV}^{2},$ | (S5) |
| --- | --- |

and given the assumption that the LV wall is 2/3 of the combined LV+SEP wall,

| $A_{m,ref,LV}=\frac{2}{3}A_{m,ref,LV+SEP} \text{and } A_{m,ref,SEP}=\frac{1}{3} A_{m,ref,LV+SEP}.$ | (S6) |
| --- | --- |

We approximated compartmental compliances (SA, SV, PA, PV), ventricular elastances (LV, RV), and intercompartmental resistances (SA, PA) in **Table A10** based on nominal pressure and volume estimates from **Table A5** or data. We assumed linear compliance. Therefore, we calculated compliance, $C$, as the ratio of the corresponding compartmental stressed volume, $V_{s}$, to the corresponding estimated systolic compartmental pressure, $\hat{P}_{M}$.

| $C_{\mathrm{SA}}=\frac{V_{SA,s}}{\hat{P}_{SA,M}}\text{, } C_{PA}=\frac{V_{PA,s}}{\hat{P}_{PA,M}}, \text{and}\text{ } C_{\mathrm{PV}}=\frac{V_{PV,s}}{\hat{P}_{PV,M}}.$ | (S7) |
| --- | --- |

$C_{\mathrm{SV}}$ was set to 3 cm^3^ kPa^-1^ for parameterization of the P21 rat. Finally, we defined SA and PA resistances as the ratio of the pressure drop across the resistance to the CO, such that

| $R_{\mathrm{SA}}=\frac{\hat{P}_{SA,M}-\hat{P}_{SV,bar}}{\mathrm{CO}}\text{ } \text{and}\text{ } R_{\mathrm{PA}}=\frac{\hat{P}_{PA,M}-\hat{P}_{PV,bar}}{\mathrm{CO}}.$ | (S8) |
| --- | --- |

**S.4 Model Variables**

The state variables for the crossbridge, myofiber, and TriSeg models are displayed in **Tables S11** and **S12**.

**Table S11. Crossbridge State Variables**

| Symbol | Definition | Unit |
| --- | --- | --- |
| $p_{1}^{0}$ | The 0th moment of state A1 strain probability distribution. Equal to the proportion of cross-bridges in state A1. | - |
| $p_{1}^{1}$ | The 1st moment of state A1 strain probability distribution. | µm |
| $p_{1}^{2}$ | The 2nd moment of state A1 strain probability distribution. | µm^2^ |
| $p_{2}^{0}$ | The 0th moment of state A2 strain probability distribution. Equal to the proportion of cross-bridges in state A2. | - |
| $p_{2}^{1}$ | The 1st moment of state A2 strain probability distribution. | µm |
| $p_{2}^{2}$ | The 2nd moment of state A2 strain probability distribution. | µm^2^ |
| $p_{3}^{0}$ | The 0th moment of state A3 strain probability distribution. Equal to the proportion of cross-bridges in state A3. | - |
| $p_{3}^{1}$ | The 1st moment of state A3 strain probability distribution. | µm |
| $p_{3}^{2}$ | The 2nd moment of state A3 strain probability distribution. | µm^2^ |
| $N$ | Non-permissible state | - |
| $U_{NR}$ | Non relaxed state | - |
| The crossbridge state variables are for each ventricular wall, RV, LV, SEP. | | |

**Table S12. Myofiber Mechanics and TriSeg State Variables**

| Symbol | Definition | Unit |
| --- | --- | --- |
| $L_{\mathrm{sc},i}$ | Contractile element length for $i=LV, SEP, RV$ | µm |
| $x_{m,LV}$ | Axial distance from origin to LV midwall surface | cm |
| $x_{m,SEP}$ | Axial distance from origin to SEP midwall surface | cm |
| $x_{m,RV}$ | Axial distance from origin to RV midwall surface | cm |
| $y_{m}$ | Radial distance from origin to RV midwall surface | cm |

**S.5 Calibrated Parameters**

The optimized parameter values from **Figure 5** are tabulated in **Table S13**. The optimized parameters separated by sex (F, M) and condition (Nx, Hx) are compared in **Table S14** and **Figure S1**.

**Table S13. Calibrated Parameters**

| Parameter | Definition | Units | Nx (n = 7) | Hx (n = 12) | p |
| --- | --- | --- | --- | --- | --- |
| $R_{\mathrm{SA}}$ | Systemic Arterial Resistance | kPa s cm^-3^ | **51.4**  ± 16.9 | **52.3**  ± 13.4 | NS |
| $R_{\mathrm{PA}}$ | Pulmonary Arterial Resistance | kPa s cm^-3^ | **16.7**  ± 6.87 | **24.3**  ± 8.85 | 0.07 |
| $A_{m,ref,LV}$ | Left Ventricular Midwall Reference Area | cm^2^ | **0.621**  ± 0.289 | **0.539**  ± 0.085 | NS |
| $A_{m,ref,SEP}$ | Septal Midwall Reference Area | cm^2^ | **0.198**  ± 0.0707 | **0.204**  ± 0.0728 | NS |
| $A_{m,ref,RV}$ | Right Ventricular Midwall Reference Area | cm^2^ | **0.724**  ± 0.134 | **1.146**  ± 0.204 | 1.5e-4* |
| $V_{w,LV}$ | Left Ventricular Wall Volume | cm^3^ | **0.0539**  ± 0.0320 | **0.0543**  ± 0.0160 | NS |
| $V_{w,SEP}$ | Septal Wall Volume | cm^3^ | **0.0492**  ± 0.0341 | **0.0245**  ± 0.0146 | 0.041* |
| $V_{w,RV}$ | Right Ventricular Wall Volume | cm^3^ | **0.0258**  ± 0.0089 | **0.0349**  ± 0.0110 | 0.08 |
| $k_{\mathrm{TS}}$ | Proportion of Cycle in Systole | - | **0.0288**  ± 0.0165 | **0.0612**  ± 0.0168 | 7.9e-4* |
| $k_{\mathrm{TR}}$ | Proportion of Cycle in Relaxation | - | **0.445**  ± 0.0335 | **0.422**  ± 0.0488 | NS |
| ***** Indicates a significant difference between Nx and Hx using a two-sided student’s t-test (p < 0.05). NS **-** not significant. | | | | | |

**Table S14. Calibrated Parameters – Separated by Sex and Condition**

| Parameter | Units | F-Nx | M-Nx | F-Hx | M-Hx | p_1_ | p_2_ |
| --- | --- | --- | --- | --- | --- | --- | --- |
| $R_{\mathrm{SA}}$ | kPa s cm^-3^ | **28.1** | **60.7**  ± 6.9392 | **46.6**  ± 7.53 | **56.4**  ± 15.7 | NS | NS |
| $R_{\mathrm{PA}}$ | kPa s cm^-3^ | **8.35** | **20.0**  ± 4.63 | **19.6**  ± 2.36 | **27.6**  ± 10.4 | NS | NS |
| $A_{m,ref,LV}$ | cm^2^ | **0.811** | **0.546**  ± 0.317 | **0.567**  ± 0.0638 | **0.520**  ± 0.0976 | NS | NS |
| $A_{m,ref,SEP}$ | cm^2^ | **0.152** | **0.217**  ± 0.0772 | **0.190**  ± 0.0844 | **0.215**  ± 0.0682 | NS | NS |
| $A_{m,ref,RV}$ | cm^2^ | **0.870** | **0.666**  ± 0.102 | **1.22**  ± 0.163 | **1.09**  ± 0.226 | NS | 0.0028* |
| $V_{w,LV}$ | cm^3^ | **0.0735** | **0.0461**  ± 0.0356 | **0.0590**  ± 0.0148 | **0.0509**  ± 0.0171 | NS | NS |
| $V_{w,SEP}$ | cm^3^ | **0.0973** | **0.0299**  ± 0.0109 | **0.0173**  ± 0.0053 | **0.0297**  ± 0.0173 | NS | NS |
| $V_{w,RV}$ | cm^3^ | **0.0292** | **0.0245**  ± 0.0103 | **0.0381**  ± 0.0142 | **0.0327**  ± 0.0085 | NS | NS |
| $k_{\mathrm{TS}}$ | - | **0.0251** | **0.0302**  ± 0.0198 | **0.0641**  ± 0.0201 | **0.0591**  ± 0.0154 | NS | 0.017* |
| $k_{\mathrm{TR}}$ | - | **0.4530** | **0.4417**  ± 0.0393 | **0.452**  ± 0.0399 | **0.402**  ± 0.0456 | NS | NS |
| p_1_ – p-values for Student’s t-test for F-Hx and M-Hx  p_2_ – p-values for Student’s t-test for M-Nx and M-Hx  ***** Indicates a significant difference using a two-sided student’s t-test (p<0.05). NS **-** not significant. | | | | | | | |

**Figure S1. Optimized Model Parameters Female (F) vs Male (M)**

**S.6 Additional Model Outputs**

Sarcomere length over the cardiac cycle is shown in **Figure S2**. Notably, RV sarcomere length is reduced in Hx compared to Nx. Pressure and volume model output signals for the Nx and Hx subjects are shown in **Figure S3** and **S4**.

**Figure S2. Ventricular Sarcomere Lengths and Shortening in Nx (green) and Hx (purple)**

**Figure S3.**

Pressure and volume signals for the Nx subjects. LV and Aortic (Ao) – Red. RV and PA – Blue. Gray lines indicate multiple heartbeats of non-filtered data. Black – average filtered data.

**Figure S4.**

Pressure and volume signals for the Hx subjects. LV and Aortic (Ao) – Red. RV and PA – Blue. Gray lines indicate multiple heartbeats of non-filtered data. Black – average filtered data.

**S.7 Additional Sensitivity and Identifiability Analysis**

Sarcomere geometry parameters and crossbridge cycling rate parameters rank as highly influential (**Figure S5**). However, we fix these parameters according to data. We restrict the parameters in contention for calibration to those at the organ-scale given that the data available in this study is at the organ-scale, as discussed in the main text. This reduces issues with parameter non-identifiability.

**Figure S5.**

Sensitivity of 54 parameters on LV and RV pressure and volume signals. The sensitivity of each parameter is normalized to each animal’s maximum sensitivity i.e. parameter sensitivity varies from 0 to 1. Parameter order is determined by average rank across all animals within the experimental group (Nx, Hx). For each animal, the corresponding normalized sensitivity is indicated by a point. The box-and-whisker plots provide the interquartile range of the normalized sensitivy for each parameter in each experimental group.

As described in **Section 3.1** of the main text, small changes in parameter values lead to differences in pressure and volume signals. Here, we present representative examples of the multistart calibration outcomes for Nx and Hx in **Figures S6** and **S7**, respectively. We see that generally parameter values cluster around the optimal parameter value, but more spread is seen in the resistance (R) and timing (k) parameters (panel **A**). Ultimately, these differences lead to substantial differences in the pressure and volume output signals (panels **B**). The spread in parameter estimates and signals suggest that the parameters are identifiable, with deviations in the final estimates due to local minima in the objective function.

**A.** **B.**

**Figure S6.**

Representative example (Nx 11) for the top 5 fits from the 20 optimized initial starts. **A.** log parameter estimates, **B.** pressure and volume solutions.

**A.** **B.**

**Figure S7.**

Representative example (Hx 1) for the top 5 fits from the 20 optimized initial starts. **A.** log parameter estimates, **B.** pressure and volume solutions.

**Appendix References**

Beneken, J. E. W. (1979). *A physical approach to hemodynamic aspects of the human cardiovascular system*. W. B. Saunders.

Boron, W. B., Emile. (2016). *Medical Physiology 3rd Edition*. ELSEVIER.

Gheorghe, A. G., Fuchs, A., Jacobsen, C., Kofoed, K. F., Møgelvang, R., Lynnerup, N., & Banner, J. (2019). Cardiac left ventricular myocardial tissue density, evaluated by computed tomography and autopsy. *BMC Med Imaging*, *19*(1), 29. <https://doi.org/10.1186/s12880-019-0326-4>

Kim, S. M., Randall, E. B., Jezek, F., Beard, D. A., & Chesler, N. C. (2023). Computational modeling of ventricular-ventricular interactions suggest a role in clinical conditions involving heart failure [Original Research]. *Frontiers in Physiology*, *14*. <https://doi.org/10.3389/fphys.2023.1231688>

Kumari, S., Braun, R. K., Tetri, L. H., Barton, G. P., Hacker, T. A., & Goss, K. N. (2019). Bimodal right ventricular dysfunction after postnatal hyperoxia exposure: implications for the preterm heart. *Am J Physiol Heart Circ Physiol*, *317*(6), H1272-h1281. <https://doi.org/10.1152/ajpheart.00383.2019>

Marzban, B., Lopez, R., & Beard, D. A. (2020). Computational Modeling of Coupled Energetics and Mechanics in the Rat Ventricular Myocardium. *Physiome*. <https://doi.org/0.36903/physiome.12964970>

Vinnakota, K. C., & Bassingthwaighte, J. B. (2004). Myocardial density and composition: a basis for calculating intracellular metabolite concentrations. *American Journal of Physiology-Heart and Circulatory Physiology*, *286*(5), H1742-H1749. <https://doi.org/10.1152/ajpheart.00478.2003>
